# Bridging ancient wisdom and cognitive engagement: a comparative study of chatbot-based moral instruction

**DOI:** 10.3389/frobt.2026.1739259

**Published:** 2026-05-11

**Authors:** Sakshi Chauhan, Varun Dutt

**Affiliations:** Applied Cognitive Science Lab, Indian Institute of Technology Mandi, Mandi, Himachal Pradesh, India

**Keywords:** chatbot-based learning, cognitive engagement, conversational AI, electroencephalography (EEG), heart rate variability (HRV), moral education

## Abstract

Convincing learners to engage deeply with complex moral and philosophical concepts remains a major challenge in contemporary learning environments, particularly within increasingly digital educational settings. Although conversational AI offers new possibilities for interactive learning, its potential for supporting ethics education remains underexplored. This study examines the effectiveness of a chatbot-based learning condition compared with a reading condition and a no-intervention control group. Learners’ outcomes were assessed through cognitive tests, self-reported emotional engagement, heart rate variability, and electroencephalographic (EEG) activity. Results showed that both the chatbot and reading conditions improved moral understanding relative to the control group. Emotional engagement was assessed during the chatbot interaction and indicated strong affective involvement among participants. EEG measures suggested increased neural engagement during the instructional conditions, while the reading condition demonstrated higher indices of attentional focus. Both intervention conditions also showed greater physiological engagement than the control group. These findings suggest that conversational AI can serve as a promising interactive tool for supporting moral learning and for facilitating deeper engagement with abstract ethical concepts in contemporary educational contexts.

## Introduction

Ethical and philosophical training still presents a significant challenge in contemporary classrooms, particularly when students are required to engage with abstract or complex texts that require sustained reflection and interpretation (Narváez and Lapsley, 2009; Kristjánsson, 2018). Teachers often encounter difficulties presenting such material in ways that remain relevant and engaging within modern digital learning environments, where students are accustomed to interactive technologies and rapid feedback cycles (Deng and Yu, 2023; Gabriella et al., 2024; Alghamdi and Holland, 2020; Zhang, 2021; Cooper and Weaver, 2003). Traditional instructional practices, including textbook reading, lectures, and explanatory instruction, often struggle to maintain students’ interest and cognitive engagement, especially among younger learners who are familiar with multimedia and technology-mediated learning environments (Khalil and Mansour, 2008). In response to these challenges, researchers and educators have increasingly explored emerging technologies such as virtual reality (VR), artificial intelligence (AI), and conversational systems to develop more engaging and personalized learning experiences that extend beyond passive information consumption (Deng and Yu, 2023). These technologies have demonstrated substantial benefits across multiple educational domains by promoting learner engagement, emotional connection, and deeper understanding through multimodal and interactive learning environments (Gabriella et al., 2024; Papamitsiou and Economides, 2014; Prieto et al., 2021). However, their application in moral education—particularly in the teaching of ethical reasoning, values, and philosophical reflection—remains relatively underexplored.

One of the most influential philosophical texts in Indian intellectual history, the Bhagavad Gita, has shaped moral and reflective thinking for centuries. The text presents a dialogical exploration of ethical dilemmas, particularly the tension between duty (dharma), personal morality, and social responsibility (Easwaran, 2007). Through the dialogue between Arjuna and Krishna, the narrative explores questions related to moral agency, justice, self-knowledge, and ethical decision-making (Easwaran, 2007). Because the text presents moral reasoning through reflective dialogue and philosophical inquiry, it has historically served as an important source for ethical instruction and philosophical reflection.

The text addresses fundamental questions of ethical responsibility, justice, self-knowledge, and the nature of consciousness, offering insights into moral decision-making and leadership (Easwaran, 2007). Despite its philosophical depth and historical significance, such classical texts remain relatively underutilized in formal educational contexts beyond cultural or religious studies. One challenge lies in the complex philosophical language and metaphorical expressions used to convey ethical ideas, which may be difficult for contemporary learners to interpret without appropriate instructional support. Previous research suggests that immersive and technology-supported storytelling methods may help bridge this gap by presenting philosophical narratives in more engaging and accessible formats (Chauhan et al., 2024a; Liu and Li, 2023; Shegaram et al., 2024; Steinhaeusser et al., 2022).

Indian moral philosophy emphasizes the development of ethical character through reflection on duty (dharma), self-awareness, and responsible action within society (Easwaran, 2007). Rather than presenting morality as a rigid set of rules, classical philosophical traditions frequently frame ethical questions through narrative dialogue and reflective inquiry. In the Bhagavad Gita, the central discussion concerns how individuals should act ethically when confronted with complex social responsibilities and internal moral conflicts (Easwaran, 2007). Such discussions encourage learners to reflect on justice, intention, responsibility, and the consequences of action. These traditions therefore align with contemporary approaches to moral education that emphasize critical reflection, perspective-taking, and ethical reasoning rather than simple memorization of moral principles (Narváez and Lapsley, 2009; Kristjánsson, 2018).

Advances in human–computer interaction have opened new possibilities for addressing these educational challenges. One promising approach is the use of conversational agents and AI-based chatbots to make philosophical content more interactive and accessible (Alghamdi and Holland, 2020; Prieto et al., 2021; Zhang, 2021; Chauhan et al., 2025; Shegaram et al., 2024). Educational chatbots based on natural language processing technologies can simulate dialogue, respond to students’ questions in real time, and provide personalized feedback, thereby supporting interactive learning processes (Deng and Yu, 2023). While such systems have been widely explored in domains such as science education, language learning, and programming instruction, their application in moral and philosophical education remains limited. This represents an important research gap, particularly given the increasing demand for culturally grounded and technology-enabled approaches to teaching ethical reasoning.

To address this gap, the present study investigates the Gitaverse Chatbot, an AI-based conversational system designed to facilitate engagement with philosophical content derived from classical Indian moral texts. Unlike general-purpose chatbots, Gitaverse is designed to guide users through philosophical ideas using interactive question-and-answer dialogue intended to simulate reflective conversation. The study includes three experimental conditions: participants who interacted with the Gitaverse Chatbot, participants who engaged with traditional reading material, and a control group with no treatment. The study examines whether the chatbot-based interaction enhances cognitive learning outcomes, emotional engagement, and physiological indicators of attentional processing, including heart rate variability (HRV) and EEG-derived neural activity measured through relative band powers (Gamma, Beta, Theta, Alpha, and Delta) as well as Alpha/Theta and Beta/Theta ratios.

From an educational perspective, moral learning involves multiple dimensions. First, it includes cognitive understanding, referring to the ability to comprehend ethical concepts and moral reasoning (Ormrod, 2016). Second, it includes affective engagement, reflecting emotional connection with moral narratives and empathy toward ethical dilemmas (Mulcahy et al., 2022; Bartels and Pecanha, 2020). Third, it involves reflective reasoning, including the capacity to interpret moral situations and evaluate alternative courses of action (Ormrod, 2016). In this study, these dimensions are operationalized through behavioral learning measures (quiz performance), self-reported emotional engagement, and physiological indicators of cognitive engagement derived from EEG and HRV measurements (Marcantoni et al., 2023; Shaffer and Ginsberg, 2017; Mulcahy et al., 2022).

By integrating classical philosophical content with contemporary AI technologies and cognitive neuroscience methods, the present research provides an interdisciplinary examination of how interactive and personalized learning environments can enhance engagement with moral and philosophical material. The study contributes empirical evidence to the emerging field of AI-mediated moral learning and offers theoretical and practical insights for designing educational tools that are technologically advanced while remaining culturally grounded.

The remainder of this paper is organized as follows. The Background Literature section reviews existing research on chatbot-based learning systems. The Methodology section describes the research design, participants, materials, and procedures. The Results section presents the behavioral, physiological, and neural findings. Finally, the Discussion section interprets the results and outlines implications for the use of conversational AI in moral education.

## Background literature

The Bhagavad Gita, which is considered one of the most influential philosophical works in classical literature, has been largely used to discuss universal ethics and moral philosophy (Easwaran, 2007). The text attempts to cover various philosophical topics such as duty, moral responsibility, and personal identity. However, the integration of such classical texts into modern education faces a number of challenges. First, the text includes complex philosophical terminologies and concepts based on ancient Sanskrit traditions and philosophical reasoning, which might be difficult for modern learners to understand (Easwaran, 2007). Secondly, the traditional way of introducing such philosophical texts to the audience involves a passive reading format, which might not be compatible with modern digital learning platforms (Easwaran, 2007). Since modern learners are accustomed to learning through various multimedia tools and interactive learning materials, the traditional way of reading philosophical texts might be quite different from their learning habits (Chauhan et al., 2024b). Therefore, there is a need to develop an effective learning approach to make philosophical dialogue more interactive and engaging for modern learners.

According to educational theories, learning can only be effective if it involves the active construction of knowledge through interaction, reflection, and dialogue. The constructivist learning theory, for example, highlights the importance of learning as an active process whereby learners are not passive recipients of knowledge, but are actively engaged in the interpretation, dialogue, and feedback process (Vygotsky and Cole, 1978). The information processing model also highlights the importance of attention, encoding, and retrieval in learning, which can be enhanced in learning environments (Atkinson and Shiffrin, 1968; Sweller, 2011). In the context of moral learning, it has long been argued that ethical learning can only be effectively facilitated if learners are engaged in reflective learning environments rather than passive learning (Dewey, 1909). More recent theories emphasize the importance of critical learning, reflective judgment, and dialogue in learning environments (Brookfield, 1998; Biesta, 2011). More recent theories also emphasize the importance of reflective dialogue and critical learning in the context of value systems (Reffhaug and Lysgaard, 2024).

All these theories suggest that learning environments involving dialogue can be very effective in facilitating learning. There are new opportunities for developing learning environments involving dialogue with the development of new learning technologies. Video learning, virtual learning environments, and chatbots, for example, have been found to be very effective in improving learning outcomes, understanding, and affective learning in various aspects of learning (Papamitsiou and Economides, 2014; Gabriella et al., 2024). For example, virtual learning environments involving narrative learning can be very effective in facilitating moral learning, which can be more effective than reading in promoting moral learning, especially if it involves rich cultural content (Chauhan et al., 2024a). Similarly, interactive learning environments involving robots can improve learning retention (Belpaeme et al., 2018). These theories are supported by the embodied cognition theories that highlight the importance of learning environments, not only involving cognitive learning but also involving the environment and emotions (Barsalou, 2008; Wilson, 2002).

Therefore, conversational AI systems, such as chatbots, are an exciting technology that could be used to develop interactive learning environments. These interactive learning environments, based on chatbots, could offer opportunities for natural dialogue, instant feedback, and tailored learning experiences. These are significant features that could be based on constructivist and dialogic learning theories, where collaborative learning and cognitive apprenticeship are key (Tegos et al., 2015). Past research on chatbot-based learning environments showed that they could be effective in improving learners’ motivation, understanding, and knowledge retention in science, coding, and language learning (Deng and Yu, 2023; Gabriella et al., 2024). Furthermore, improvements in conversational interfaces, based on advances in natural language processing, could be used to offer contextual feedback, thus encouraging learners to engage more deeply in learning materials, even in situations where the materials are cognitively demanding. However, despite the interest in developing educational chatbots, their use in moral and philosophical learning, particularly in relation to classical philosophical literature, is an area that has been little explored.

These conversational learning environments, based on dialogue and collaborative learning, could also have an effect on learners’ emotional engagement with philosophical narratives. Emotional engagement is an essential aspect in moral reasoning, and learners’ empathic and emotional responses are essential in dealing with moral dilemmas.

Following these theoretical perspectives, this current research investigates how various modalities of instruction affect emotional engagement, cognitive learning outcomes, and physiological engagement in a philosophical learning task. This research addresses the following research questions: How does interaction with a conversational AI system affect learners’ emotional engagement in a philosophical learning task in comparison to a traditional reading task? Do chatbot-based and reading-based instructional modalities vary in terms of their effect on moral comprehension outcomes? How do various instructional modalities affect physiological engagement in a moral learning task? Do conversational AI-based and reading-based instructional modalities elicit unique neural engagement in a philosophical learning task?

Interactive conversational systems may elicit greater emotional engagement in a learning task because they mimic a dialogic conversation with a human partner and allow a learner to actively ask and engage with a subject. Therefore, it is expected that:

Hypothesis 1 Participants who interact with the Gitaverse Chatbot will report greater emotional engagement in a learning task in comparison to participants in a reading task.

Interactive systems may also affect cognitive outcomes in a learning task. The constructivist model of learning suggests that dialogic conversation in an interactive system will increase conceptual understanding because it elicits a learner to actively engage with a subject by asking questions. However, it is also expected that traditional philosophical content will elicit greater moral comprehension in a learner in comparison to a control group that does not engage in a traditional instructional task. Therefore, it is expected that:

Hypothesis 2 Participants in a Gitaverse Chatbot task and a reading task will elicit greater moral comprehension outcomes in comparison to a control group.

Hypothesis 3 Participants in a Gitaverse Chatbot task will elicit greater moral comprehension outcomes in comparison to participants in a reading task.

Recent studies on educational neuroscience have also highlighted the role of physiological measures of engagement while learning. Heart rate variability (HRV), specifically the Root Mean Square of Successive Differences (RMSSD), is used as a physiological measure of parasympathetic nervous activity and cognitive–emotional engagement. A decrease in the value of RMSSD while engaging in cognitively demanding tasks is often linked with high levels of autonomic arousal and mental effort, which is a sign of engagement with the material. Therefore, if the learners are engaged with the material, their physiological measures should reveal high levels of cognitive effort compared to those who might not be engaged with the material. Thus, we expect:

Hypothesis 4 The values of the participants in the Gitaverse Chatbot and reading conditions are expected to be lower after the intervention in comparison to the control condition, reflecting increased cognitive and emotional engagement in the learning activity.

EEG is an electrophysiological measurement that uses neural activity to measure cognitive engagement in the brain. It measures oscillatory activity in different frequency bands. In cognitive neuroscience studies, theta activity is often related to cognitive control and memory integration, and alpha and beta activity are related to attentional control and cognitive engagement (Marcantoni et al., 2023). The ratios of alpha and beta activity, i.e., Alpha/Theta and Beta/Theta, are often related to sustained attentional processing in structured learning tasks (Halderman, 2021). Gamma activity is often related to integrative cognitive processing in reasoning tasks (Marcantoni et al., 2023). The nature of conversational learning involves interpreting, questioning, and integrating the responses, and thus it might be related to neural activity that is different from that in reading tasks. So, the research hypothesis is:

Hypothesis 5 The neural engagement in the instructional conditions will vary as inferred by the EEG activity. The Gitaverse Chatbot condition is expected to have increased levels of theta and gamma activity, reflecting cognitive and interactive engagement, and the reading condition is expected to have increased levels of Alpha/Theta and Beta/Theta, reflecting increased sustained attentional processing.

In addition to its empirical contribution to understanding the effect of a particular educational technology, this study also makes a theoretical contribution by considering the effect of conversational learning environments on cognitive, affective, and neural processes. By incorporating a conversational AI with both physiological (HRV) and neural (EEG) measures, this study offers a multi-modal approach to understanding the effect of interactive learning technologies on engagement with complex philosophical material.

In order to test these hypotheses, this study will investigate the effect of the Gitaverse Chatbot, a document-informed conversational learning environment that incorporates a classical philosophical text. The chatbot will be used to simulate a dialogic learning experience by providing learners with the ability to ask questions related to the philosophical material. This type of conversational learning environment will allow learners to engage with complex philosophical material by interpreting the material through a dialogue with the chatbot.

In order to evaluate the effect of these learning conditions, this study will compare the effect of interaction with the Gitaverse Chatbot, reading a philosophical text, and a no-intervention control group on both cognitive learning outcomes (quiz performance), affective learning outcomes (self-reported emotional responses), and physiological learning outcomes (HRV and EEG). This multi-modal approach will allow this study to provide a multi-modal approach to understanding the effect of interactive learning technologies on engagement with complex philosophical material.

To the best of our knowledge, this study will be one of the first to investigate the effect of a chatbot-based moral learning environment informed by classical philosophical texts on both behavioral learning outcomes, emotional engagement, physiological learning outcomes (HRV), and neural learning outcomes (EEG).

## Methods

### Research design

The objective of the study was to examine the relative effect of three instructional conditions—the Gitaverse Chatbot condition, the reading condition, and the control condition on cognitive and affective engagement with moral lessons derived from a classical philosophical reading.

**FIGURE 1:**
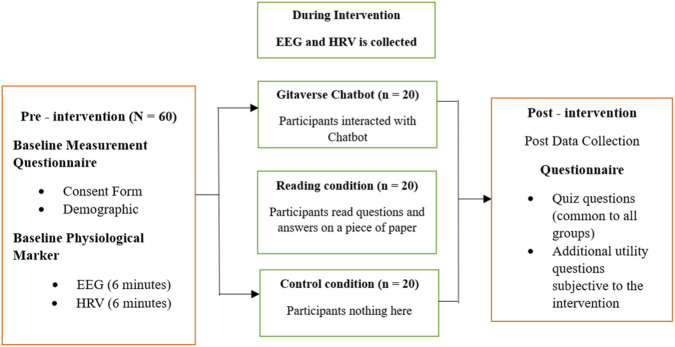
Experimental design of the study. Participants were randomly assigned to one of three instructional conditions: Gitaverse Chatbot interaction, traditional reading, or a no-intervention control condition. The experiment consisted of three phases: pre-intervention (demographic questionnaire and baseline HRV measurement), intervention (7-min instructional exposure with continuous HRV and EEG recording), and post-intervention (comprehension quiz and emotional engagement survey for the chatbot group).

As shown in Figure 1, the study compared three instructional conditions. A total of 60 participants were randomly assigned to one of three conditions: the Gitaverse Chatbot condition, the reading condition, or the no-intervention control condition (n = 20 per condition). Participants were not informed about the existence of multiple experimental conditions. Participants were told that the study examined learning and responses to educational material; however, the specific hypotheses regarding differences between instructional modalities were not disclosed. The study followed a double-blind design in which both participants and the experimenters involved in administering the sessions and collecting data were unaware of the specific research hypotheses and experimental comparisons between conditions.

In the Gitaverse Chatbot condition, participants interacted with an AI chatbot by typing pre-prepared questions related to the reading material. In the reading condition, participants were exposed to the same material presented in a paper-based question-and-answer format without interactive dialogue. The control condition did not receive any instructional content but remained seated for the same duration as the intervention conditions to maintain comparable experimental timing across conditions.

Behavioral and physiological data were collected to examine participants’ responses to the intervention. Behavioral data included moral comprehension assessed through quiz responses. Physiological data included heart rate variability (HRV) and electroencephalography (EEG) indicators of cognitive engagement.

As shown in Figure 1, the experiment was conducted across three phases: pre-intervention, intervention, and post-intervention. During the pre-intervention phase, participants in all conditions completed a demographic questionnaire and underwent baseline HRV assessment. During the intervention phase, participants in the Gitaverse Chatbot and reading conditions individually received the stimulus material, while physiological activity was monitored for all conditions for 7 minutes. Participants in the control group remained seated quietly for the same 7-min period without receiving instructional material. During this interval, EEG and HRV were recorded continuously for all participants. Participants were instructed to remain seated and refrain from interacting with researchers or engaging in other activities in order to maintain consistent experimental conditions. During the post-intervention phase, participants completed a multiple-choice test assessing knowledge retention, and participants in the chatbot group additionally completed an emotional engagement survey.

### Participants

Table 1 presents the demographic characteristics of participants across the three instructional conditions. A total of 60 participants were recruited and randomly assigned to one of three experimental conditions: Gitaverse Chatbot, reading, or control (n = 20 per condition). Participants were university students aged between 18 and 30 years across all conditions. In the Gitaverse Chatbot group, the mean age was 21.35 years (*Median* = 21, *SD* = 2.10), with 80% male and 20% female participants. In the reading group, the mean age was 21.05 years (*Median* = 21, *SD* = 1.63), with 85% male and 15% female participants. In the control group, the mean age was 21.05 years (*Median* = 21, *SD* = 2.50), with 80% male and 20% female participants.

**TABLE 1:** Demographic characteristics of participants across the three instructional conditions (Gitaverse Chatbot, reading, and control). Values represent counts, percentages, and descriptive statistics for each group.

Characteristic	Gitaverse chatbot (n = 20)	Reading (n = 20)	Control (n = 20)
Age			
Mean	21.35	21.05	21.05
Median	21	21	21
SD	2.10	1.63	2.50
Gender			
Male N (%)	16 (80%)	17 (85%)	16 (80%)
Female N (%)	4 (20%)	3 (15%)	4 (20%)
Academic background			
Computer science (%)	88.89%	90%	90%
Electronics engineering (%)	5.56%	5%	0%
Data science (%)	5.56%	5%	0%
Computational and signal processing (%)	0%	0%	5%
Electrical engineering (%)	0%	0%	1%
Compensation	₹40 (≈$0.52)	₹40 (≈$0.52)	₹40 (≈$0.52)

Participants were enrolled primarily in engineering and technology-related academic programs, including Computer Science and Engineering, Electronics Engineering, Data Science, and Computational and Signal Processing. The predominance of engineering students reflects the institutional context in which the study was conducted and provides a relevant population for examining technology-mediated moral learning. Engineering education increasingly emphasizes ethical reasoning, responsible technology development, and awareness of the societal implications of artificial intelligence systems (National Academies of Sciences, Engineering, and Medicine, 2020; Shuman et al., 2005; IEEE, 2019). Investigating chatbot-based moral learning within this population therefore offers insight into how interactive AI tools may support ethical reflection among technically oriented learners (Fiesler et al., 2020; Saltz et al., 2018). Participation in the study was voluntary, and each participant received compensation of INR 40 (approximately USD 0.52).

### Stimuli

The stimulus material was derived from Chapter 1 of the Bhagavad Gita, an ancient Indian philosophical text, using the English translation by Eknath Easwaran (Easwaran, 2007). The selected passage presents themes of justice and ethical responsibility, central concepts in the moral discourse of the text (Easwaran, 2007).

The intervention was implemented through two primary intervention conditions: the Gitaverse Chatbot group and the reading group. Participants in both conditions engaged with the same textual material derived from Chapter 1 of the Bhagavad Gita; however, the mode of delivery differed. The Gitaverse Chatbot group interacted with the material through an LLM-based chatbot interface, whereas the reading group received the same content through a conventional reading format. Thus, the instructional content remained constant across conditions, while the modality of delivery constituted the experimental manipulation.

### Gitaverse Chatbot

As shown in Figure 1, those in the Gitaverse Chatbot condition engaged in conversation with a reflective character, grounded in content from an original philosophical text.

The chatbot was driven by OpenAI’s GPT-3.5 language model (OpenAI, 2023) and augmented by retrieval-augmented generation (RAG) in an effort to leverage responses based on the source text. This was an effort to create higher levels of understanding of the Gita’s philosophical issues, e.g., duty (dharma), moral dilemma, and the nature of the self—through an individualized and interactive process.

**FIGURE 2:**
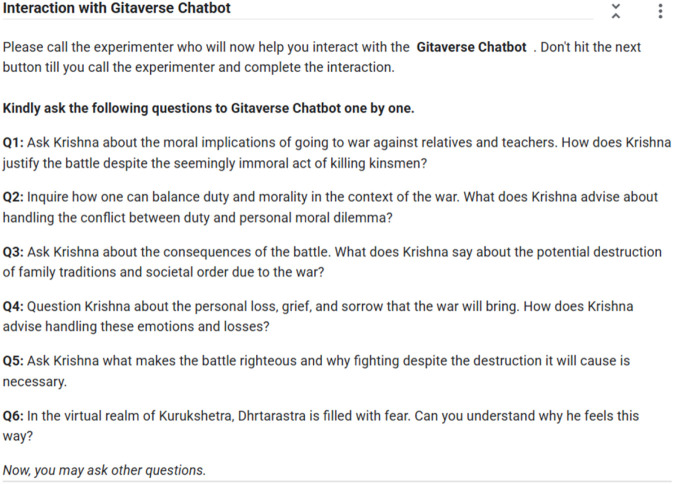
Predefined question set used in the Gitaverse Chatbot condition. Participants entered these questions into the chatbot interface to initiate dialogue about philosophical concepts derived from the Bhagavad Gita.

**FIGURE 3:**
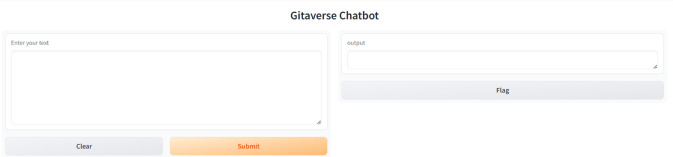
Gitaverse Chatbot user interface used during the experiment. Participants entered questions into the input field and received generated responses from the conversational AI system grounded in the philosophical source text.

Participants were presented with a predetermined set of questions (Figure 2), which participants typed into the web-based input of the chatbot. The input interface, as shown in Figure 3, allowed participants to type questions into a text box with the phrase “Enter your text.” Upon clicking the “Submit” button, the chatbot gave immediate feedback, with the feedback being presented in the output field. This format was designed to mimic a Socratic dialogue format, thereby making the ancient scripture more readable to users who utilize conversational exchange.

### Development of document-aware Chatbot

**FIGURE 4:**
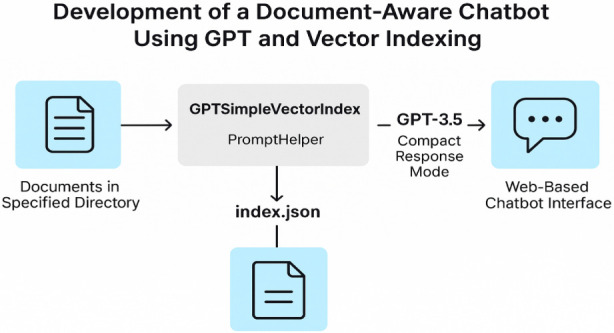
System architecture for the document-aware Gitaverse Chatbot. The chatbot integrates GPT-3.5 with retrieval-augmented generation using LangChain and LlamaIndex. Source documents from the Bhagavad Gita were indexed and retrieved during user queries to generate contextually grounded responses.

A chatbot was created to offer an interactive learning environment based on Indian Knowledge Systems (IKS). It was built using OpenAI’s GPT-3.5 model and integrated with LangChain and LlamaIndex (previously GPT Index) (OpenAI, 2023; LangChain, 2023; LlamaIndex, 2024; see Figure 4). The goal was to allow learners to interact with a classical philosophical text using natural language so that they could receive responses that were contextually relevant, comprehensible, and grounded in the source material.

The back-end system employed GPTSimpleVectorIndex to index Gita texts stored locally in a directory. The index enabled the chatbot to retrieve pertinent passages in response to student queries. A PromptHelper instance managed model constraints and chunk sizes to support efficient interaction within the GPT-3.5 system. Once the documents were indexed, they were stored in an index. json file, which facilitated efficient retrieval in subsequent interactions.

When a participant submitted a question, the chatbot searched the indexed content, retrieved relevant segments, and generated a response using OpenAI’s language model in compact response mode. The interface was constructed using Gradio (Abid et al., 2021), which offered a clean and intuitive experience for both technical and non-technical users.

This architecture enabled learners to access ancient moral philosophy through an interactive conversational interface and illustrates how conversational AI can support moral reflection, cognitive engagement, and deeper learning. By turning the Gita into an interactive digital environment, the Gitaverse Chatbot helped bridge ancient knowledge and contemporary educational technology, enabling students to engage with challenging ethical ideas in an accessible, flexible, and meaningful way.

### Reading group

Participants in the reading group received the same core moral content as those in the Gitaverse Chatbot group, but in a static text-based format derived from the same philosophical source. They were presented with a printed page containing seven pre-formulated questions and matching answer choices. The printed Q&A form was structured to match the chatbot material so that participants across conditions received the same philosophical content. Unlike the chatbot condition, however, the reading group did not receive interactivity or adaptive feedback.

### Comprehensive quiz questions

As shown in Table 2, the seven multiple-choice items assessed participants’ familiarity with major moral and philosophical principles presented in Chapter 1 of the Bhagavad Gita, including the nature of the soul, duty (dharma), justice (adharma), inner conflict, and righteous action. The first item examined learners’ understanding of the eternal nature of the soul, which conceptually supports psychological detachment and may contribute to reduced mental stress when confronting life challenges (Q1). The second item assessed understanding of the warrior’s duty to uphold justice and righteousness, reflecting schema-based moral reasoning about social responsibility (Q2). The third item addressed the tension between personal moral dilemmas and duty, encouraging cognitive decentering by prompting learners to consider how individuals resolve inner conflict (Q3). The fourth item evaluated comprehension of Krishna’s central teaching regarding duty and the impermanence of life, which engages higher-order reasoning about ethical decision-making during crisis (Q4). The fifth item focused on Dhritarashtra’s fear in the context of dharmakshetra, highlighting situational awareness and ethical anticipation in moral judgment (Q5). The sixth item examined beliefs about those who die fighting for righteousness, reinforcing motivational cognition related to moral commitment and purpose (Q6). Finally, the seventh item assessed understanding of what makes a battle righteous, emphasizing principle-based reasoning grounded in the restoration and preservation of dharma (Q7). Collectively, the items were designed to elicit moral reasoning, emotional regulation, and reflective evaluation of ethical principles.

**TABLE 2:** Moral comprehension quiz questions, answer options, and correct responses. Seven multiple-choice items assessing participants’ understanding of philosophical concepts from Chapter 1 of the Bhagavad Gita, including duty (dharma), moral conflict, justice, and the nature of the soul.

S.No.	Question	Options	Correct answer	Rationale
1	Q1. What does Krishna explain about the nature of the soul?	a) The soul is mortal and dies with the body b) The soul is eternal and simply changes bodies c) The soul is fragmented and dispersed after death d) The soul ceases to exist after death e) None of these	b	Learners understands detachments leading to less mental stress
2	Q2. What is the warrior’s duty according to Krishna?	a) To avoid war at all costs b) To protect relatives, even if it means neglecting duty c) To fight for justice and righteousness d)To surrender to the enemy e) None of these	c	The concept enforces schema-based moral reasoning
3	Q3. How does Krishna suggest handling the conflict between duty and personal moral dilemma?	a) Prioritize personal moral dilemma over duty b) Balance between duty and moral dilemma c) Fulfill the warrior duty to fight irrespective of personal conflict d)Abandon duty to avoid moral conflict e) None of these	a	Illustrates cognitive decentering, encouraging learners to resolve inner conflict
4	Q4. What crucial lesson does Krishna teach Arjuna in Chapter 1 of the Bhagavad Gita when Arjuna is in crisis?	a) The importance of wealth and power b) The importance of duty and the impermanence of life c) The importance of personal happiness d)The importance of surrender and giving up e) None of these	b	Helps in developing higher order reasoning
5	Q5. Why was Dhrtarastra fearful in the Bhagavad Gita Chapter 1?	a) He was afraid of the might of the Pandavas’ army b) He feared the influence of Kurukshetra as a dharmakshetra, favoring the victory of the Pandavas c)He doubted his own sons’ capabilities in battle d) He was afraid of Lord Krishna’s power e) None of these	b	Reflects situational awareness and ethical anticipation
6	Q6. What does Krishna say about those who die in the battle fighting for righteousness?	a) They are forgotten b) They are condemned c)They reach heavenly abodes d) They are reborn as lower beings e) None of these	c	Reinforces motivational cognition
7	Q7. What makes the battle righteous according to Krishna?	a) The number of warriors involved b) The skills and weapons used c) The restoration and upholding of dharma (righteousness) d) The vengeance against the enemy e) None of these	c	Highlights principle-based reasoning

### Control group

The control group did not receive any exposure to the material derived from the traditional philosophical text. In this condition, participants only completed the physiological measures and demographic questionnaires during the pre-intervention phase. During the intervention phase, participants remained seated quietly while HRV and EEG data were recorded to provide a baseline physiological comparison.

At post-intervention, all three conditions, including the control group, answered the same multiple-choice test. The control condition’s responses were used as a baseline measure of familiarity and performance without exposure, providing a comparison against which to assess the effect of content-based interventions in the other two conditions.

### HRV measurement and sensor details

The emWave Pro biofeedback equipment, designed by the HeartMath Institute, was utilized for the collection of Heart Rate Variability (HRV) data in the present study (HeartMath Institute, 2016). The equipment is based on the measurement of HRV using photoplethysmography (PPG) sensors, which are non-invasive sensors connected to the participant’s earlobe or finger. The sensors are able to detect the changes in the volume of the pulse in the blood, thus enabling the measurement of the inter-beat intervals, which correspond to the rhythm of the heartbeats. After the collection of the HRV data, the R-R interval (RR) was exported in. txt format for further analysis. The HRV data was then analyzed using specialized software for the analysis of HRV, enabling the measurement of the autonomic nervous system. Specifically, the Root Mean Square of Successive Differences (RMSSD) was utilized as the HRV measurement, as it is recognized as a reliable method of measuring the autonomic nervous system and the activity of the parasympathetic nervous system and cognitive-emotional activity (Shaffer and Ginsberg, 2017). The HRV measurement of RMSSD has been utilized in various educational and cognitive research studies for the assessment of the physiological activity in relation to attention, mental effort, and emotional involvement in learning tasks (Mulcahy et al., 2022; Shaffer and Ginsberg, 2017). The utilization of the emWave Pro biofeedback equipment for the measurement of HRV was thus based on the non-invasive measurement of physiological engagement in the moral learning interventions.

### EEG measurement and neural data processing

Neural activity was recorded using the MUSE S EEG headband (InteraXon Inc.), an affordable, wearable EEG headset that was validated for cognitive neuroscience research (Krigolson et al., 2017). It has four dry electrodes: two placed at frontal positions (AF7, AF8) and two at temporal sides (TP9, TP10). EEG activity was recorded at 256 Hz, and a 50 Hz notch filter was used prior to data acquisition to reduce power line noise.

Real-time signal checking was used during setup to verify signal quality across all channels. Motion artifacts such as eye blinks, jaw clenching, and muscle activity were reduced during preprocessing, and a Butterworth filter, commonly used in EEG preprocessing, was applied (Ghassemi et al., 2018). Preprocessing ensured that clean neural data were retained for frequency-domain analysis.

### Relative band power generation

Relative band powers were computed to determine the participants’ cognitive abilities in response to the intervention provided. Thus, raw data are taken from the Muse S file, and the raw data from the Muse band are obtained in the (0–1,682 μV) range, whereas brain signals work in the (−100–100 μV) range (Krigolson et al., 2017). From this raw file, four columns were selected (RAW TP9, RAW AF7, RAW AF8, and RAW TP10) and first converted into the (−100–100 µV) range. After bringing down to this range, the Butterworth Filter is applied to remove any artifacts such as jaw clenches and eye blinks. After applying the filter, the fast Fourier transform was used to collect data for various frequency components, such as delta waves (0.5–4 Hz), beta waves (13–30 Hz), theta waves (4–8 Hz), alpha waves (8–13 Hz), and gamma waves (30–100 Hz).

Upon obtaining the absolute value of the power bands of all five brain signals, the relative power was calculated by applying the following formula (see Equation 1):

$$Relative\;x=\frac{\sum_{ch=1}^{4}\;Absolute\;Power_{x,ch}}{\sum_{ch=1}^{4\;}\sum_{b=1}^{5\;}Absolute\;Power_{Band\;b,ch}}$$ (1)

Here, Relative x reflects the relative contribution of a specific frequency band to global neural activity rather than its absolute magnitude. Absolute Power_(x,ch)_ denotes the absolute spectral power of frequency band x (delta, theta, alpha, beta, or gamma) measured at channel *ch*, where *ch* = 1 … 4. The numerator represents the sum of absolute power in the selected frequency band across all four EEG channels. The denominator corresponds to the total absolute spectral power summed across all five canonical frequency bands and across all four channels. The resulting ratio therefore yields the relative power of band *x*, normalized by the total spectral power across all bands and channels, providing a global proportionate measure of band-specific activity independent of overall signal magnitude.

### Data analysis

Quiz responses, emotional engagement ratings, usability ratings, and HRV metrics (RMSSD) were analyzed using conventional inferential statistical procedures. Quiz performance was scored as the proportion of correct responses out of seven items. Emotional engagement and usability satisfaction were measured using self-report Likert-scale items ranging from 1 (strongly disagree) to 5 (strongly agree), consistent with common psychometric practice (Boone and Boone, 2012). Physiological involvement was quantified with the root mean square of successive differences (RMSSD), a time-domain HRV parameter of great reliability applied widely to predict parasympathetic nervous system activity and emotional-cognitive involvement (Shaffer and Ginsberg, 2017).

For HRV analyses, a two-way repeated-measures ANOVA was conducted with intervention group (reading, chatbot, control) as the between-subjects factor and time (pre-intervention, post-intervention) as the within-subjects factor. Main effects and interaction effects were examined for RMSSD values.

For behavioral outcomes, group differences in quiz scores were analyzed using one-way ANOVA, followed by Tukey’s Honestly Significant Difference (HSD) *post hoc* comparisons where appropriate. Emotional engagement and usability responses were summarized descriptively because these measures were collected only in the chatbot condition.

Where significant main or interaction effects were observed, *post hoc* comparisons were conducted using Tukey’s Honestly Significant Difference (HSD) test to control for family-wise error (Field, 2024). Statistical significance was evaluated at p < 0.05 in all analyses. An *a priori* statistical power level of 0.80 was used as the minimum acceptable threshold (Cohen, 2013).

### Behavioral results

#### Correct average quiz score across questions

**FIGURE 5:**
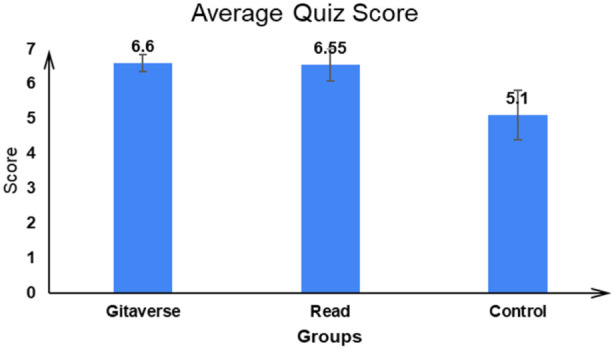
Mean quiz performance across instructional conditions. Average number of correct responses (out of seven) for participants in the Gitaverse Chatbot, reading, and control conditions. Error bars represent standard errors.

The average results for participants in the three conditions—Gitaverse Chatbot, reading, and control—across seven items are displayed in Figure 5. The correct average score obtained was considerably different, as shown in Figure 5, *F* (2, 57) = 12.25, *p* < 0.01, *η* ^2^ = 0.31. A significant difference was found using the Tukey *post hoc* test between the reading and control group (read: μ = 6.55 > control: μ = 5.10 (*p* < 0.01)) and the Gitaverse Chatbot and control group (Gitaverse Chatbot: μ = 6.60 > control: μ = 5.10 (*p* < 0.01)). But there was no significant difference between the reading and Gitaverse Chatbot conditions (read: μ = 6.55 ∼ Gitaverse Chatbot: μ = 6.60 (p = 0.98)). Overall, these findings support Hypothesis 2, indicating that both the Gitaverse Chatbot and reading conditions produced higher moral comprehension scores than the control condition, while the absence of a significant difference between the chatbot and reading conditions suggests comparable learning outcomes, partially aligning with Hypothesis 3.

#### Influence of demographic variables on behavioral outcomes

To examine whether demographic variables influenced behavioral outcomes, an ANCOVA model was conducted including age, gender, and field of study as covariates. None of the demographic variables significantly predicted quiz performance (age: *F* (1, 43) = 0.031, *p* = 0.861; gender: *F* (1, 43) = 0.001, *p* = 0.971; education: *F* (2, 43) = 0.322, *p* = 0.727). The effect of instructional condition remained non-significant after controlling for these variables (*F* (2, 43) = 2.403, *p* = 0.103). No significant interaction effects between instructional condition and demographic variables were observed.

#### Emotional engagement with Gitaverse Chatbot

**FIGURE 6:**
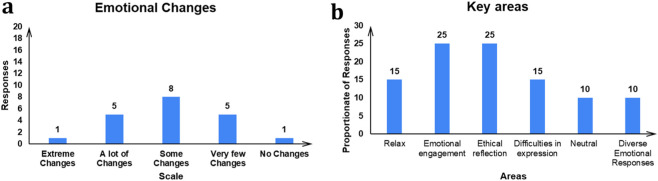
Emotional engagement reported in the Gitaverse Chatbot condition. **(a)** Distribution of participants’ self-reported emotional or physiological changes during chatbot interaction. **(b)** Thematic categories derived from qualitative responses describing emotional and cognitive experiences during the chatbot interaction.

Participants’ responses to the question “Did you notice any changes in your emotional or physiological state during interacting with the Gitaverse Chatbot about Bhagavad Gita?” was analyzed (see Figure 6a). Figure 6a shows the Likert scale values from “Extreme changes” to “No changes”. As shown in Figure 6a, one participant felt “Extreme changes”, five participants felt “A lot of changes”, eight participants felt “Some changes”, five participants felt “Very few changes”, and only 1 participant felt “No changes”. Whereas, Figure 6b, shows the key areas obtained from the responses against the question “If you did feel changes in your emotional or physiological state, please describe any feelings or sensations you experienced”. The major areas obtained were: Relaxation and Calmness, Cognitive and emotional engagement, Ethical and moral reflection, Difficulties in expression, Neutral, Relation, Perspective shift (see Figure 6b). All things considered, these responses indicate meaningful emotional and reflective engagement during the chatbot interaction, supporting Hypothesis 1, which predicted that participants interacting with the Gitaverse Chatbot would report stronger emotional engagement with the learning material due to the conversational and responsive nature of the chatbot environment.

## HRV data: RMSSD

**FIGURE 7:**
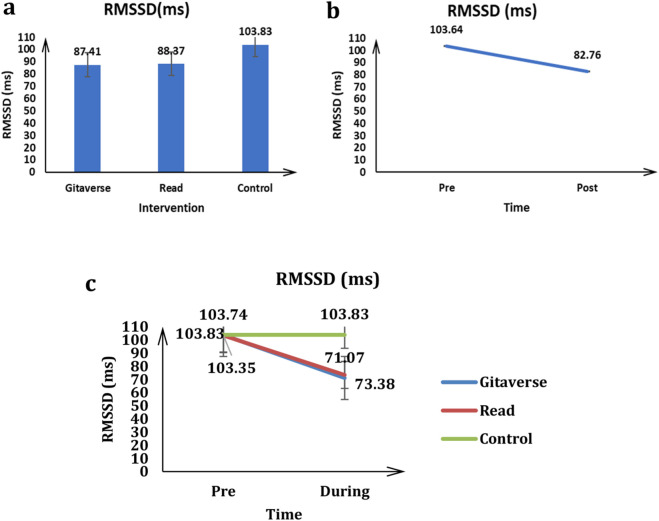
Heart rate variability (RMSSD) results across instructional conditions. **(a)** Main effect of instructional condition on RMSSD values. **(b)** Main effect of time (pre-intervention vs. post-intervention). **(c)** Interaction between instructional condition and time.

The main effect of the group (Gitaverse Chatbot, reading and control) on the RMSSD was significant, *F* (2, 57) = 3.96, *p* < 0.05, *η* ^2^ = 0.12 (see Table 3). The control (μ = 103.83) had a higher RMSSD value than the Gitaverse (μ = 87.41) and read (μ = 88.37) conditions (see Figure 7a). The effect of time was significant, *F* (2, 57) = 32.87, *p* < 0.001, *η* ^2^ = 0.48 (see Figure 7b). The pre-time measurement (μ = 103.64) had a higher RMSSD value than the post-time measurement (μ = 82.76). The interaction between conditions (Gitaverse Chatbot, reading and control) and time was significant, *F* (2, 57) = 8.26, *p* < 0.001, *η* ^2^ = 0.22 (see Figure 7c). As shown in Figure 7c, at the pre-time measurement, the RMSSD value was similar between the Gitaverse Chatbot (μ = 103.74), read (μ = 103.35), and the control (μ = 103.83). However, at the post-time measurement, the RMSSD value of the control group (μ = 103.83) was higher than reading (μ = 73.38, *p* < 0.05), and Gitaverse Chatbot (μ = 71.07, *p* < 0.05). All things considered, these findings support Hypothesis 4, indicating that participants in the Gitaverse Chatbot and reading conditions exhibited lower RMSSD values following the intervention compared to the control group, reflecting increased cognitive and emotional engagement during the learning activity.

**TABLE 3:** Descriptive statistics and repeated-measures ANOVA results for HRV (RMSSD). Mean ± standard error values are reported for each instructional condition before and after the intervention, along with the main effects of time, group, and the interaction between time and instructional condition.

Effect	Gitaverse (mean ± SE)		Reading (mean ± SE)		Control (mean ± SE)		Time effect	Group effect	Interaction effect
	Pre	Post	Pre	Post	Pre	Post	*F*(*p*, *η* ^2^)	*F*(*p*, *η* ^2^)	*F*(*p*, *η* ^2^)
RMSSD	103.74 ± 6.23	71.07 ± 4.89	103.35 ± 6.23	73.38 ± 4.89	103.35 ± 6.23	103.83 ± 4.89	32.87 (<0.001, 0.48)	3.96 (<0.05, 0.12)	8.26 (<0.001, 0.22)

### Neural data

#### Alpha/theta ratio

**FIGURE 8:**
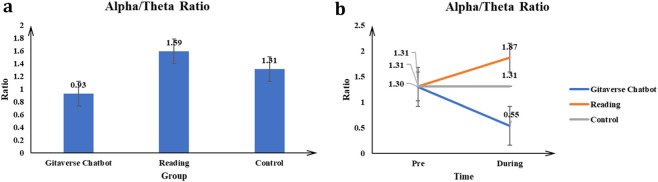
Alpha/Theta ratio results across instructional conditions. **(a)** Main effect of instructional condition on Alpha/Theta ratio. **(b)** Interaction effect between instructional condition and time.

The main effect of the conditions (Gitaverse Chatbot, reading, and control) on the Alpha/Theta Ratio was significant *F* (2, 57) = 30.30, *p* < 0.001, *η* ^2^ = 0.52 (see Figure 8a; Table 4). Post-hoc tests indicated reading (μ = 1.59) had a higher Alpha/Theta Ratio than the Gitaverse Chatbot (μ = 0.93), and control (μ = 1.31) conditions (see Figure 8a). The interaction between conditions (Gitaverse Chatbot, reading, and control) and time was significant, *F* (2, 57) = 29.65, *p* < 0.001, *η* ^2^ = 0.51 (see Figure 8b). As shown in Figure 8b, at the pre-time measurement, the Alpha/Theta Ratio was similar between the Gitaverse Chatbot (μ = 1.30), reading (μ = 1.31), and the control (μ = 1.31). However, at the post-time measurement, the Alpha/Theta Ratio of the reading (μ = 1.87) was higher than Gitaverse Chatbot (μ = 0.55) and control (μ = 1.31). The effect of time was not significant, *F* (1, 57) = 0.90, *p* = 0.35, *η* ^2^ = 0.02. Overall, these findings are consistent with Hypothesis 5, suggesting modality-related differences in neural engagement, with the reading condition showing stronger Alpha/Theta ratios associated with sustained attentional focus during the learning task.

**TABLE 4:** Descriptive statistics and repeated-measures ANOVA results for EEG measures. Mean ± standard error values for Alpha/Theta ratio, Beta/Theta ratio, and relative spectral band powers (delta, theta, alpha, beta, gamma) across instructional conditions before and after the intervention.

EEG metric	Gitaverse (mean ± SE)		Reading (mean ± SE)		Control (mean ± SE)		Time effect	Group effect	Interaction effect
	Pre	Post	Pre	Post	Pre	Post	*F*(*p*, *η* ^2^)	*F*(*p*, *η* ^2^)	*F*(*p*, *η* ^2^)
Alpha/Theta ratio	1.304 ± 0.01	0.545 ± 0.231	1.311 ± 0.01	1.870 ± 0.231	1.310 ± 0.01	1.480 ± 0.231	0.90 (0.35, 0.02)	30.30 (<0.001, 0.52)	29.65 (<0.001, 0.51)
Beta/Theta ratio	1.256 ± 0.009	0.597 ± 0.167	1.261 ± 0.009	1.825 ± 0.167	1.189 ± 0.009	1.149 ± 0.167	0.12 (0.73, 0.002)	15.62 (<0.001, 0.35)	15.52 (<0.001, 0.35)
Relative delta	0.216 ± 0.01	0.199 ± 0.019	0.215 ± 0.000	0.253 ± 0.019	0.213 ± 0.01	0.243 ± 0.019	1.03 (0.32, 0.01)	5.55 (<0.001, 0.16)	5.80 (<0.001, 0.17)
Relative theta	0.196 ± 0.006	0.264 ± 0.014	0.190 ± 0.006	0.155 ± 0.014	0.195 ± 0.006	0.195 ± 0.014	2.30 (0.14, 0.04)	11.42 (<0.001, 0.29)	16.94 (<0.001, 0.37)
Relative alpha	0.206 ± 0.01	0.132 ± 0.018	0.205 ± 0.01	0.253 ± 0.018	0.203 ± 0.01	0.243 ± 0.018	2.88 (>0.05, 0.04)	46.73 (<0.001, 0.62)	48.25 (<0.001, 0.63)
Relative beta	0.208 ± 0.005	0.133 ± 0.012	0.207 ± 0.005	0.232 ± 0.012	0.207 ± 0.005	0.207 ± 0.012	7.08 (<0.001, 0.11)	12.74 (<0.001, 0.31)	22.88 (<0.001, 0.44)
Relative gamma	0.21 ± SE	0.27 ± SE	0.21 ± SE	0.15 ± SE	0.22 ± SE	0.22 ± SE	0.00 (>0.05, 0.00)	10.06 (<0.001, 0.26)	9.35 (<0.001, 0.24)

#### Beta/theta ratio

**FIGURE 9:**
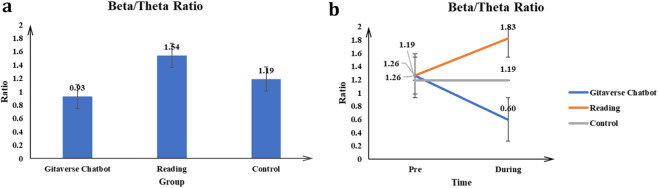
Beta/Theta ratio results across instructional conditions. **(a)** Main effect of instructional condition on Beta/Theta ratio. **(b)** Interaction effect between instructional condition and time.

The main effect of the conditions (Gitaverse Chatbot, reading, and control) on the Beta/Theta Ratio was significant *F* (2, 57) = 15.62, *p* < 0.001, *η* ^2^ = 0.35 (see Table 4). Post-hoc tests indicated reading (μ = 1.54) had a higher Beta/Theta Ratio than the Gitaverse Chatbot (μ = 0.93), and control (μ = 1.19) conditions (see Figure 9a). The interaction between conditions (Gitaverse Chatbot, reading, and control) and time was significant, *F* (2, 57) = 15.52, *p* < 0.001, *η* ^2^ = 0.35 (see Figure 9b). As shown in Figure 9b, at the pre-time measurement, the Beta/Theta Ratio was similar between the Gitaverse Chatbot (μ = 1.26), reading (μ = 1.26), and the control (μ = 1.19). However, at the post-time measurement, the Beta/Theta Ratio of the reading (μ = 1.83) was higher than Gitaverse Chatbot (μ = 0.60) and control (μ = 1.19). The effect of time was not significant, *F* (1, 57) = 0.12, *p* = 0.73, *η* ^2^ = 0.002. Overall, these findings are consistent with Hypothesis 5, suggesting that the reading condition elicited stronger Beta/Theta ratios associated with sustained attentional processing during the learning task.

#### Relative delta

**FIGURE 10:**
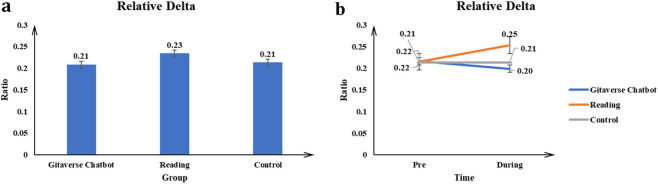
Relative delta power across instructional conditions. **(a)** Main effect of instructional condition on relative delta activity. **(b)** Interaction effect between instructional condition and time.

The main effect of the conditions (Gitaverse Chatbot, reading, and control) on the Relative Delta value was significant *F* (2, 57) = 5.55, *p* < 0.001, *η* ^2^ = 0.16 (see Table 4). Post-hoc tests indicated reading (μ = 0.23) had a higher Relative Delta value than the Gitaverse Chatbot (μ = 0.21), and control (μ = 0.21) conditions (see Figure 10a). The interaction between conditions (Gitaverse Chatbot, reading, and control) and time was significant, *F* (2, 57) = 5.80, *p* < 0.001, *η* ^2^ = 0.17 (see Figure 10b). As shown in Figure 10b, at the pre-time measurement, the Relative Delta value was similar between the Gitaverse Chatbot (μ = 0.21), reading (μ = 0.22), and the control (μ = 0.22). However, at the post-time measurement, the Relative Delta value of the reading (μ = 0.25) was higher than the Gitaverse Chatbot (μ = 0.20) and control (μ = 0.21). The effect of time was not significant, F (1, 57) = 1.03, *p* = 0.32, *η* ^2^ = 0.01. Overall, these findings contribute to the neural engagement patterns predicted in Hypothesis 5, indicating modality-related differences in EEG activity across the instructional conditions.

#### Relative theta

**FIGURE 11:**
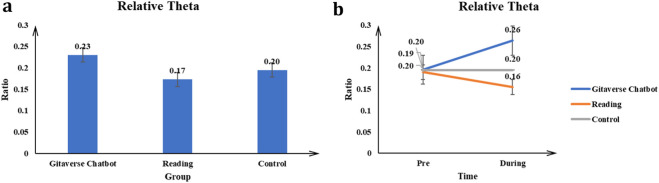
Relative theta power across instructional conditions. **(a)** Main effect of instructional condition on relative theta activity. **(b)** Interaction effect between instructional condition and time.

The main effect of the conditions (Gitaverse Chatbot, reading, and control) on the Relative Theta value was significant *F* (2, 57) = 11.42, *p* < 0.001, *η* ^2^ = 0.29 (see Table 4). Post-hoc tests indicated Gitaverse Chatbot (μ = 0.23) had a higher Relative Theta value than the reading (μ = 0.17), and control (μ = 0.20) conditions (see Figure 11a). The interaction between conditions (Gitaverse Chatbot, reading, and control) and time was significant, *F* (2, 57) = 16.94, *p* < 0.001, *η* ^2^ = 0.37 (see Figure 11b). As shown in Figure 11b, at the pre-time measurement, the Relative Theta value was similar between the Gitaverse Chatbot (μ = 0.20), reading (μ = 0.19), and the control (μ = 0.20). However, at the post-time measurement, the Relative Theta value of the Gitaverse Chatbot (μ = 0.26) was higher than the reading (μ = 0.16) and control (μ = 0.20). The effect of time was not significant, F (1, 57) = 2.30, *p* = 0.14, *η* ^2^ = 0.04. Overall, these findings align with Hypothesis 5, suggesting that the chatbot condition elicited higher relative theta activity associated with interactive cognitive engagement during the learning task.

#### Relative alpha

**FIGURE 12:**
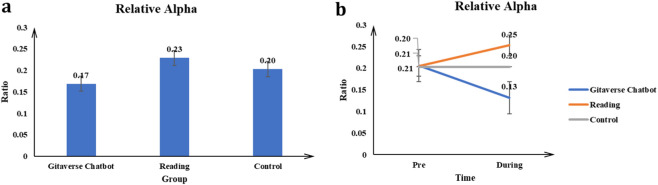
Relative alpha power across instructional conditions. **(a)** Main effect of instructional condition on relative alpha activity. **(b)** Interaction effect between instructional condition and time.

The main effect of the conditions (Gitaverse Chatbot, reading, and control) on the Relative Alpha value was significant *F* (2, 57) = 46.73, *p* < 0.001, *η* ^2^ = 0.62 (see Table 4). Post-hoc tests indicated reading (μ = 0.23) had a higher Relative Alpha value than the Gitaverse Chatbot (μ = 0.17), and control (μ = 0.20) conditions (see Figure 12a). The interaction between conditions (Gitaverse Chatbot, reading, and control) and time was significant, *F* (2, 57) = 48.25, *p* < 0.001, *η* ^2^ = 0.63 (see Figure 12b). As shown in Figure 12b, at the pre-time measurement, the Relative Alpha value was similar between the Gitaverse Chatbot (μ = 0.21), reading (μ = 0.21), and the control (μ = 0.20). However, at the post-time measurement, the Relative Alpha value of the reading (μ = 0.25) was higher than the Gitaverse Chatbot (μ = 0.13) and control (μ = 0.20). The effect of time was not significant, *F* (1, 57) = 2.88, *p* > 0.05, *η* ^2^ = 0.04. Because relative alpha activity was not explicitly stated in Hypothesis 5, these findings should be treated as supplementary evidence consistent with stronger attentional engagement in the reading condition, rather than as a direct test of the hypothesis.

#### Relative beta

**FIGURE 13:**
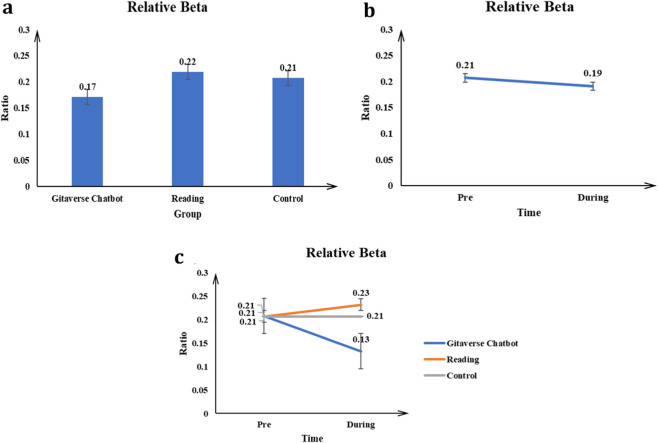
Relative beta power across instructional conditions. **(a)** Main effect of instructional condition on relative beta activity. **(b)** Main effect of time (pre-vs. post-intervention). **(c)** Interaction effect between instructional condition and time.

The main effect of the conditions (Gitaverse Chatbot, reading, and control) on the Relative Beta value was significant *F* (2, 57) = 12.74, *p* < 0.001, *η* ^2^ = 0.31 (see Table 4). Post-hoc tests indicated reading (μ = 0.22) had a higher Relative Beta value than the Gitaverse Chatbot (μ = 0.17), and control (μ = 0.21) conditions (see Figure 13a). The effect of time was significant, *F* (1, 57) = 7.08, *p* < 0.001, *η* ^2^ = 0.11 (see Figure 13b). The pre-time measurement (μ = 0.21) had a higher Relative Beta value than the post-time measurement (μ = 0.19). The interaction between conditions (Gitaverse Chatbot, reading, and control) and time was significant, *F* (2, 57) = 22.88, *p* < 0.001, *η* ^2^ = 0.44 (see Figure 13c). As shown in Figure 13c, at the pre-time measurement, the Relative Beta value was similar between the Gitaverse Chatbot (μ = 0.21), reading (μ = 0.21), and the control (μ = 0.21). However, at the post-time measurement, the Relative Beta of the reading (μ = 0.23) was higher than Gitaverse Chatbot (μ = 0.13), and control (μ = 0.21). Because Hypothesis 5 specifically referred to Beta/Theta ratio rather than relative beta power alone, these findings should be interpreted as supplementary neural evidence consistent with stronger attentional processing in the reading condition.

#### Relative gamma

**FIGURE 14:**
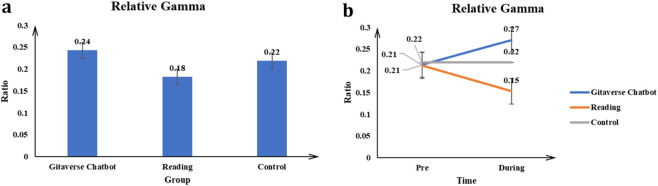
Relative gamma power across instructional conditions. **(a)** Main effect of instructional condition on relative gamma activity. **(b)** Interaction effect between instructional condition and time.

The main effect of the conditions (Gitaverse Chatbot, reading, and control) on the Relative Gamma value was significant *F* (2, 57) = 10.06, *p* < 0.001, *η* ^2^ = 0.26 (see Table 4). Post-hoc tests indicated Gitaverse Chatbot (μ = 0.24) had a higher Relative Gamma value than the reading (μ = 0.18), and control (μ = 0.22) conditions (see Figure 14a). The interaction between conditions (Gitaverse Chatbot, reading, and control) and time was significant, *F* (2, 57) = 9.35, *p* < 0.001, *η* ^2^ = 0.24 (see Figure 14b). As shown in Figure 14b, at the pre-time measurement, the Relative Delta value was similar between the Gitaverse Chatbot (μ = 0.21), reading (μ = 0.21), and the control (μ = 0.22). However, at the post-time measurement, the Relative Gamma value of the Gitaverse Chatbot (μ = 0.27) was higher than the reading (μ = 0.15) and control (μ = 0.22). The effect of time was not significant, *F* (1, 57) = 0.00, *p* > 0.05, *η* ^2^ = 0.01. This pattern supports the Chatbot-related gamma component of Hypothesis 5. Therefore, Hypothesis 5 was partially supported overall: the Chatbot condition showed higher theta and gamma activity, whereas the reading condition showed higher Alpha/Theta and Beta/Theta ratios.

## Discussion

This study investigated whether conversational artificial intelligence can support moral learning from classical philosophical texts. Specifically, the study compared three instructional conditions, Gitaverse Chatbot interaction, traditional reading, and a passive control condition to examine differences in cognitive learning outcomes, emotional engagement, physiological responses, and neural activity. Overall, the results indicate that both the Chatbot-based and reading-based instructional formats significantly enhanced participants’ understanding of moral concepts relative to passive exposure. These findings provide empirical evidence that structured exposure to philosophical material—whether through conversational interaction or traditional reading—can support meaningful engagement with ethical concepts in digitally mediated learning environments. At the same time, distinct patterns of physiological and neural engagement emerged across the two instructional modalities, suggesting that different learning formats may activate different cognitive processes during moral learning.

The behavioural results demonstrate that exposure to structured moral content significantly improved participants’ comprehension and retention of philosophical concepts. Both the Gitaverse Chatbot (M = 6.60) and reading (M = 6.55) conditions produced significantly higher quiz scores than the control condition (M = 5.10). These findings support theories of cognitive learning which emphasize that active engagement with educational material promotes deeper encoding and retrieval of information (Ormrod, 2016). In the present study, both instructional formats required participants to process moral concepts, reflect on ethical dilemmas, and integrate philosophical ideas presented in the Bhagavad Gita. Thus, the findings support Hypothesis 2, indicating that exposure to structured philosophical content—irrespective of modality—can enhance learners’ comprehension of moral concepts.

Interestingly, the Chatbot condition produced slightly higher comprehension scores than the reading condition, although the difference was not statistically significant. This outcome partially supports Hypothesis 3 and suggests that conversational learning environments may offer modest benefits for conceptual understanding, although these advantages may depend on the nature of the task and the complexity of the learning material. Previous research on educational chatbots has shown that dialogic interfaces can promote conceptual clarification by enabling learners to ask questions, receive immediate feedback, and engage in iterative reasoning processes (Tegos et al., 2015; Deng and Yu, 2023; Gabriella et al., 2024). These findings also align with constructivist learning theory, which suggests that knowledge is constructed through interaction, reflection, and feedback rather than passive information reception (Vygotsky and Cole, 1978). In the context of moral education, such dialogic interaction may help learners explore ethical dilemmas more actively than static reading formats.

Participants interacting with the Gitaverse Chatbot reported a range of emotional and reflective experiences while engaging with the moral content. Qualitative responses suggested feelings of calm reflection, ethical contemplation, and perspective shifts during the chatbot interaction. These findings provide support for Hypothesis 1 and indicate that conversational AI systems may promote affective engagement with philosophical narratives, an important component of moral reasoning and ethical reflection.

Research in affective computing and adaptive learning systems suggests that interactive systems capable of responding dynamically to user input can create learning environments that feel socially responsive and cognitively engaging. Conversational agents may therefore promote deeper emotional involvement by simulating dialogue, a pedagogical form historically associated with philosophical inquiry. Classical philosophical traditions—including Socratic dialogue and dialogic reasoning in Indian philosophical texts—often rely on conversational structures to facilitate ethical reflection. By recreating such dialogic engagement in a digital environment, the Gitaverse Chatbot provides a technologically mediated form of philosophical dialogue that may help learners engage more deeply with ethical questions.

At the same time, emotional engagement was measured only in the chatbot condition in the present study. Therefore, while the results suggest meaningful affective engagement during conversational learning, future research should directly compare emotional responses across instructional modalities to better understand how different learning environments influence affective engagement with ethical material.

Physiological data provided additional insight into learners’ engagement during the instructional tasks. HRV analyses revealed lower RMSSD values in both the chatbot and reading conditions compared with the control condition. Lower RMSSD values during cognitive tasks have been associated with increased autonomic arousal and greater attentional engagement (Shaffer and Ginsberg, 2017; Mulcahy et al., 2022). These findings support Hypothesis 4 and suggest that both instructional modalities required active cognitive effort, reflecting heightened physiological engagement during the processing of philosophical material.

These physiological findings complement the behavioural results by indicating that active processing of moral content requires sustained cognitive effort. Importantly, the similarity between the chatbot and reading conditions suggests that engagement with philosophical material itself—regardless of delivery format—can activate cognitive and emotional processes involved in ethical reasoning.

Neural data revealed modality-specific patterns of cognitive processing. The reading condition demonstrated higher Alpha/Theta and Beta/Theta ratios, as well as increased relative alpha and beta activity. These neural patterns are commonly associated with sustained attentional processing and structured cognitive effort during reading tasks (Marcantoni et al., 2023). These results partially support Hypothesis 5 and suggest that traditional reading promotes sustained attentional focus and structured cognitive processing when learners engage with complex textual material.

In contrast, the Gitaverse Chatbot condition exhibited higher relative theta and gamma activity. Theta oscillations are often linked to cognitive control, memory integration, and reflective processing, while gamma activity has been associated with integrative reasoning and higher-order cognitive processing (Marcantoni et al., 2023). The increased theta and gamma activity observed during chatbot interaction therefore suggests that conversational learning environments may encourage integrative reasoning processes associated with reflective dialogue and iterative interpretation.

Taken together, these neural patterns suggest that the two instructional modalities engage different cognitive pathways. Reading appears to promote sustained attentional processing, whereas conversational AI interaction may facilitate more integrative and interactive cognitive engagement. These findings align with theories of embodied and interactive cognition, which propose that learning emerges through dynamic interaction between the learner, the environment, and the cognitive system (Barsalou, 2008; Wilson, 2002).

The findings of this study have several implications for the design of technology-enhanced moral education. First, the results suggest that classical philosophical texts can be effectively integrated into digital learning environments when presented through structured and accessible formats. Both reading and chatbot-based instruction improved comprehension of moral concepts, indicating that exposure to carefully structured ethical content can support meaningful learning outcomes.

Second, conversational AI systems may provide unique opportunities for engaging learners with complex philosophical ideas. By simulating dialogic interaction, chatbots can transform traditional texts into interactive learning experiences that encourage reflection and inquiry. This capability may be particularly valuable for philosophical education, where dialogue and interpretation play central roles in understanding ethical principles.

Third, the findings are particularly relevant in the context of engineering and technology education. Modern engineering curricula increasingly emphasize ethical reasoning, responsible innovation, and awareness of the societal implications of artificial intelligence and emerging technologies. Within this context, conversational AI systems such as the Gitaverse Chatbot may provide a useful pedagogical tool for encouraging ethical reflection among technically oriented learners. By integrating philosophical reasoning with technology-mediated learning environments, such systems may help engineering students engage with questions of responsibility, fairness, and societal impact that are increasingly central to contemporary technological practice.

Finally, the study demonstrates the potential of combining educational technology with physiological and neural measurement methods to gain deeper insight into learning processes. Such multimodal approaches may provide valuable tools for evaluating how different instructional designs influence cognitive engagement in educational settings.

Although the present findings are promising, several limitations should be considered. First, the participant sample consisted primarily of university students aged 18–30 years, with a predominance of male participants and a concentration of engineering and technology majors. While this population provides a relevant context for studying AI-mediated ethical learning among technically oriented learners, the results may not fully generalize to other academic or demographic groups. Future research should therefore examine more diverse participant populations—including students from humanities, social sciences, and interdisciplinary programs—to determine whether similar engagement patterns emerge across different educational backgrounds.

Second, emotional engagement was measured only in the chatbot condition. Future studies should include comparable affective measures across multiple instructional conditions to enable direct comparisons of emotional engagement between conversational and non-interactive learning formats.

Third, the present study focused on short-term comprehension and engagement outcomes. Moral learning often involves longer-term reflection and value formation. Future research should therefore examine the long-term impact of conversational AI on ethical reasoning, moral decision-making, and value internalization.

Finally, future studies may explore hybrid instructional designs that combine conversational AI with immersive technologies such as virtual reality or adaptive storytelling environments. Such approaches could further enhance engagement with philosophical content by integrating dialogue, narrative immersion, and experiential learning.

## Conclusion

In summary, this study demonstrates the potential of conversational AI as a tool for facilitating engagement with complex moral and philosophical ideas. Both chatbot-based and reading-based instructional formats significantly improved learners’ comprehension of moral concepts relative to passive exposure. At the same time, the two instructional modalities elicited distinct physiological and neural engagement patterns, suggesting that conversational AI and traditional reading activate different cognitive processes during moral learning.

Importantly, the results highlight the potential role of conversational AI in supporting ethical reflection within technology-oriented educational contexts. As engineering and computing disciplines increasingly confront questions related to responsible innovation, AI governance, and societal impact, educational tools that promote ethical reasoning may become increasingly important.

The Gitaverse Chatbot represents a promising approach for bridging classical philosophical knowledge with contemporary educational technology. By transforming traditional philosophical texts into interactive dialogue-based learning experiences, conversational AI systems may help learners engage more deeply with ethical concepts and reflective reasoning. More broadly, integrating culturally grounded philosophical traditions with emerging AI technologies may open new directions for interdisciplinary research at the intersection of cognitive science, education, and human-centered artificial intelligence.

## Significance statement

Ethical education, particularly when derived from classical texts, tends to find it challenging to engage contemporary learners who are more and more surrounded by digital and interactive learning settings. Conventional practices like passive reading might not promote the requisite profound cognitive and affective engagement to internalize abstract moral ideas. This research addresses an increasing demand in education: how to effectively teach philosophical thought in ways that speak to today’s learners. We examined whether an interactive chatbot, trained on content from a traditional philosophical text, would enhance cognitive and affective engagement compared to reading and no-intervention conditions. Building on real-world difficulties in teaching ethics and waning attention in traditional learning mediums, our research integrates AI-facilitated conversation with cognitive science approaches-applying quiz scores, heart rate variability (HRV), and EEG responses to estimate learning and engagement. The findings indicated that students who conversed with the chatbot not only scored better on understanding tasks but also manifested more intense physiological and neural markers of engagement. These results indicate that conversational AI can be an effective bridge between ageless philosophical insights and modern cognitive learning requirements. By anchoring the intervention in both ancient material and recent technology, this study provides an applied and theory-informed method for rejuvenating moral education for the digital age.

## Data Availability

The raw data supporting the conclusions of this article will be made available by the authors, without undue reservation.
